# Physical properties of a sandy soil as affected by incubation with a synthetic root exudate: Strength, thermal and hydraulic conductivity, and evaporation

**DOI:** 10.1111/ejss.13007

**Published:** 2020-06-28

**Authors:** Wencan Zhang, Weida Gao, William Richard Whalley, Tusheng Ren

**Affiliations:** ^1^ Department of Soil and Water Sciences China Agricultural University Beijing China; ^2^ Department of Sustainable Agricultural Sciences Rothamsted Research Harpenden UK

**Keywords:** heat conduction, microbial activity, soil penetrometer resistance, water retention

## Abstract

Plant roots release various organic materials that may modify soil structure and affect heat and mass transfer processes. The objective of this study was to determine the effects of a synthetic root exudate (SRE) on penetrometer resistance (PR), thermal conductivity (λ), hydraulic conductivity (*k*) and evaporation of water in a sandy soil. Soil samples, mixed with either distilled water or the SRE, were packed into columns at a designated bulk density and water content, and incubated for 7 days at 18°C. Soil PR, λ, *k* and evaporation rate were monitored during drying processes. Compared with those incubated with water, samples incubated with SRE had visible hyphae, greater PR (0.7–5.5 MPa in the water content range of 0.11 to 0.22 m^3^ m^−3^) and λ (0.2–0.7 W m^−1^ K^−1^ from 0.05 to 0.22 m^3^ m^−3^), and increased *k* in the wet region but decreased *k* in the dry region. SRE treatment also reduced the overall soil water evaporation rate and cumulative water loss. Analysis of X‐ray computed tomography (CT) scanning showed that the SRE‐treated samples had a greater proportion of small pores (<60 μm). These changes were attributed mainly to SRE‐stimulated microbial activities.

**Highlights:**

- The effects of incubating a sandy soil with a synthetic root exudate (SRE) on soil physical properties and evaporation are examined.
- SRE incubation increased the fraction of small pores.
- SRE incubation increased soil penetrometer resistance and thermal conductivity.
- Soil hydraulic conductivity was increased in the wet region but was reduced in the dry region.
- SRE incubation reduced the overall evaporation rate and cumulative water loss.

## 1 INTRODUCTION

Secretion or exudation from plant roots and microbes, often termed mucilage or exudates, affects the structure and physical properties of soils (Barré & Hallett, 2009; Carminati et al., 2010; Choudhury, Ferraris, Ashton, Powlson, & Whalley, 2018; Czarnes, Hallett, Bengough, & Young, 2000; Lambers, Mougel, Jaillard, & Hinsinger, 2009; Read et al., 2003; Whalley et al., 2005). Mucilage, a matrix of higher‐molecular‐weight compounds (Sasse, Martinoia, & Northen, 2018), is polysaccharide‐rich and can behave as a viscoelastic gel. Exudates are composed of a wide range of components, including mucilage and other low‐molecular‐weight or soluble high‐molecular‐weight compounds (Galloway, Knox, & Krause, 2020). Holz, Carminati, and Kuzyakov (2015) found that root exudates and mucilage have a high degree of spatial structure with distance from the root tip and with radial distance from the root. The chemical compounds in natural root exudates depend on many factors, such as the plant species and age and soil microbial communities and activity, as well as the soil chemical and physical status (Naveed et al., 2017).

The organic compounds in root exudates play an important role in the plant–microbe interaction by affecting soil microbial communities’ structure and function (Shi et al., 2011), and the utilization of this type of carbon is an important driver of soil microbial abundance. Soil microbial activity and community development is often stimulated or determined by natural root exudates (Paterson, Gebbing, Abel, Sim, & Telfer, 2007) or synthetic root exudates (Choudhury et al., 2018; Gao et al., 2017). The changes in microbial community or microbial activity will in turn alter soil physical properties. Gao et al. (2017) found that microbial activity was stimulated by a low‐molecular synthetic root exudate, and that a poikilothermic temperature response of soil penetrometer resistance seemed to be related to fungi or *Streptomyces*, a filamentous bacterium. As temporary binding agents, fungal hyphae can bond microaggregates (<0.25 mm diameter) into stable macroaggregates (>0.25 mm diameter) (Tisdall, 1991). Root exudates also modify soil structure by altering aggregate stability, strength and particle contacts (Carminati et al., 2010; Czarnes et al., 2000; Hinsinger, Bengough, Vetterlein, & Young, 2009; Read et al., 2003; Whalley et al., 2005). For example, there are reports that root exudates or mucilage increased soil aggregate stability (Morel, Habib, Plantureux, & Guckert, 1991; Traoré, Groleau‐Renaud, Plantureux, Tubeileh, & Boeuf‐Tremblay, 2000). After adding polygalacturonic acid (PGA) and xanthan, soil strength and stability were increased following wetting and drying cycles, which was attributed to the increased particle bonding strength by PGA (Czarnes et al., 2000). Similarly, some researchers observed that addition of PGA led to an increase of bonding energy among soil particles (Zhang, Hallett, & Zhang, 2008), and enhanced soil hardness and modulus of elasticity, and that the degree of the changes varied with the source of the exudates (Naveed et al., 2017). Soil structure can be affected by root exudates per se, specifically their high viscosity and low surface tension (Gregory, 2006; Lavelle, 2002; Strayer, Power, Fagan, Pickett, & Belnap, 2003; Watt, Silk, & Passioura, 2006; Hinsinger et al., 2009).

The changes of soil structure in the rhizosphere caused by root exudates may alter soil hydraulic properties and processes. Zhang et al. (2008) observed a decrease of evaporation rate and an increase of soil water retention, which were attributed to the extracellular polymeric substances (EPS) produced by a plant growth‐promoting rhizobacteria. There are reports that root exudates can both increase soil water holding capacity (Nakanishi, Okuni, Hayashi, & Nishiyama, 2005; Read, Gregory, & Bell, 1999; Young, 1995) and reduce water contents in the rhizosphere (Dunbabin, McDermott, & Bengough, 2006; Read et al., 2003; Whalley et al., 2005). An increase in soil water repellence has also been reported (Carminati et al., 2010; Moradi et al., 2012). These conflicting findings with respect to water retention are more likely to be explained by the different types of compounds secreted by roots, and some polymeric gels (e.g., glucose) are generally present to increase water holding capacity, whereas some surfactants (e.g., phospholipid) act to decrease water holding capacity (Read et al., 2003). Drying is an important process and it highlights the structural difference between rhizosphere and bulk soil. For example, Nambiar (1976) and Watt, McCully, and Canny (1994) observed the formation of a more stable maize rhizosphere in the drier soils, Albalasmeh and Ghezzehei (2014) reported that the drying process enhanced soil aggregation by transporting and depositing binding agents, such as PGA and xanthan, and Benard et al. (2017) pointed out that root exudates were deposited preferentially in small pores during drying. A micromorphological study showed an increase in porosity of mucilage‐treated soil following wetting and drying cycles (Czarnes et al., 2000).

Heat transfer, available water, nutrient contents, aeration and soil strength are important factors that determine both microbial activity and root growth. Soil thermal conductivity (λ) is a parameter that characterizes soil heat conduction. It is determined by the volumetric proportions of solid, liquid and gaseous phases, the arrangement of soil particles, and the interfacial contact between the solid particles as well as between the solid and liquid phases (Carson et al., 2003; Farouki, 1986). It is likely that microbial activity that alters pore size distribution or particle bonding will also affect λ, but to our knowledge this has never been studied. Soil penetrometer resistance (PR), which is commonly used to characterize the soil strength and resistance to root penetration, depends on soil structure and water status (Bengough, Campbell, & OÄôSullivan, 2000; Vaz, Manieri, de Maria, & Tuller, 2011), particle size, shape and distribution, and the chemistry of the soil solution (Horn, 1994). Gao et al. (2017) observed an increase of PR in soil samples incubated with a synthetic root exudate. Evaporation of water from a bare soil surface depends on the hydraulic connectivity of soil and it is widely accepted that this can be modified by the activity of both roots and microbes.

In brief, root exudates alter the rhizosphere soil structure and water holding capacity, but it is unclear how other soil physical properties and processes respond to these changes. In this study we investigate the changes of soil PR, λ, *k* and evaporation following the addition of a synthetic root exudate, which is known to stimulate microbial activity (Choudhury et al., 2018; Gao et al., 2017). The effect of root exudate on soil pore size distribution was also examined with X‐ray computed tomography (CT). This is the first report of the integrated effects of a root exudate on thermal, hydraulic and mechanical properties of the rhizosphere.

## 2 MATERIALS AND METHODS

### 2.1 Soil sample and synthetic root exudate

The soil sample was collected in May 2017 from the surface layer (0–20 cm) of a maize field located in Lishu County of Jilin Province, China. The soil has a sand texture with 92% sand, 3% silt and 5% clay. After removing crop residues and fine roots, the partly air‐dried soil sample (with a water content about 0.07 g g^−1^) was passed through a 2‐mm sieve and then stored in the dark at 4°C.

A synthetic root exudate (SRE) solution was prepared by mixing 15 compounds, including five large molecular polysaccharides (glucose, fructose, sucrose, arabinose and ribose), five amino acids (glycine, valine, glutamine, serine and alanine) and five organic acids (malic, citric, malonic, oxalic and fumaric), following the procedures of Paterson et al. (2007). Each compound contributed 1.39 g C in 500 mL distilled water, giving a solution with 4.167% C and 7.34% N.

### 2.2 Soil column preparation and incubation experiment

To examine the effects of microbial activity on soil PR, λ, *k*, water evaporation rate and soil pore size distributions with X‐ray CT, we prepared soil columns with SRE‐treated and distilled water‐treated samples (control). The sieved soil sample (with initial water content of about 0.07 g g^−1^) was mixed with either synthetic root exudate or distilled water at a concentration of 6 ml solution 100 g^−1^ dry soil, which gave a water content of 0.13 g g^−1^ (close to field capacity). This concentration was equivalent to 7.2 mg SRE g^−1^ dry soil, which was greater than the natural exudate concentration (4.6 mg exudate g^−1^ dry soil) reported by Zickenrott et al. (2016), but was within the range (4 and 8 mg g^−1^ particle) of Benard et al. (2019).

For PR, λ and pore structure measurements, paired soil samples (mixed with SRE and water) were packed into polycarbonate pipes (50 mm high and 50 mm internal diameter [i.d.]) with a 200 kPa axial pressure (equivalent to the stress of a tractor), which gave an approximate bulk density of 1.61 g cm^−3^. A total of 40 soil columns (20 for each treatment) were prepared. These samples were incubated in the dark for 7 days at a temperature of 18°C. After incubation, 34 samples (17 for each treatment) were placed in a temperature‐regulated room (25±1°C) and subjected to air‐drying to investigate the changes of PR and λ with water content: 30 samples (five times and three reps for each treatment) for PR measurement and four samples (two reps for each treatment) for monitoring λ continuously. The remaining six samples (three reps for each treatment) were air‐dried and used to examine the pore structure with X‐ray CT scanning.

To examine the effects of SRE addition on soil water evaporation rate and hydraulic conductivity, six larger soil columns (about 61 mm high, 72 mm i.d. and a volume of 250 cm^3^, triplicate samples for each treatment) were prepared in the same way as the smaller columns and incubated in the dark for 7 days at 18°C.

### 2.3 Measuring soil evaporation and hydraulic conductivity

The six larger samples were saturated with distilled water and placed on a ku‐pF apparatus (DT 04‐01, Umwelt‐Geräte‐Technik GmbH, Müncheberg, Germany). The equipment has a star‐shaped revolving table that has 10 sample holders, each carrying one sample ring and two tensiometers. The distance between the top and bottom tensiometers is 3 cm. Sample weights were recorded with an automatically lowering and lifting balance at 1‐hr intervals. Soil water evaporation rates were calculated from the dynamics of weight losses. The hydraulic conductivity *k*, was calculated by using Equation (1):

() $$k=\frac{∆V}{2A∆t}\frac{\Delta z}{\left(\Psi_{t}-\Psi_{b}\right)-\Delta h},$$

where *∆V* is the volume of evaporative water loss during time *∆t*, *A* is the cross‐sectional area of the sample ring, *Ψ*_*t*_ is the tension of the top tensiometer (as positive pressure in the unsaturated range), *Ψ*_*b*_ is the tension of the bottom tensiometer, *Δz* is the distance between the tensiometers in the sample ring (=3 cm), and *Δh* is the altitude difference between the tensiometer positions (=3 cm).

### 2.4 Measuring soil penetrometer resistance

The PRs of soil samples were measured with a universal testing machine with a measuring range of 0–100 N and an accuracy of 0.00001 N (Model UTM6102, Shenzhen Suns Technology Stock Co., Ltd, Shenzhen, China). The machine was equipped with a 2‐mm diameter and 60° cone angle penetrometer. The penetrometer was inserted into soil samples at a constant speed of 20 mm min^−1^ (Gao et al., 2012; Moraes et al., 2019) from the surface to a depth of 45 mm (to protect the pressure transducer from exceeding maximum load), and the PR was recorded automatically. After PR measurements, the samples were oven‐dried at 105°C for more than 24 hr to determine bulk density and water content. The PR measurements were conducted periodically during a 5‐day drying period.

### 2.5 Measuring soil thermal conductivity

We used a three‐needle heat‐pulse probe (40‐mm long and 1.3‐mm‐diameter stainless steel waveguides with 6‐mm spacing) to measure λ (Ren et al., 1999). The probe was inserted into the soil column vertically. A constant current was applied to the central needle (which included a heater) for 7–10 s to generate the heat pulse. A data logger (CR 3000, Campbell Scientific, Logan, UT, USA) recorded the temperature change at the sensing needles and the power input at a 1‐s interval for 300 s. For each sample, the measurement was repeated three times at a 60‐min time interval. The temperature‐by‐time data were analysed using the single‐point method (Bristow, Bilskie, Kluitenberg, & Horton, 1995; Bristow, Kluitenberg, & Horton, 1994).The daily λ measurements were continued for 8 days until the water contents approached a relatively stable state. At the end of the drying process, soil bulk density and water content were determined by oven‐drying the samples at 105°C for more than 24 hr.

### 2.6 X‐ray CT scanning and image processing

The soil cores (three for each treatment) were scanned with an industrial Phoenix Nanotom X‐ray μ‐CT (GE, Sensing and Inspection Technologies, GmbH, Wunstorf, Germany) at an energy of 100 kV and a current of 100 μA. A 0.2‐mm Cu filter was used to reduce the beam hardening effect. The filtered back‐projection algorithm was used to reconstruct slices from the radiographs. About 2,000 slices with a size of 2,000 × 2,000 pixel for each slice were reconstructed for every sample. The final slices were in 16‐bit format, with a resolution of 10 μm.

Image processing, visualization and quantification were carried out with an open source software Image J ver. 1.51 (Rasband, 1997–2011). Images were first imported, changed to 8‐bit format and adjusted for brightness. We used a global threshold segmentation method to segment the images and the threshold selection was carefully chosen based on the visual observation method of Zhou, Peng, Peth, and Xiao (2012). A region of interest (ROI) of 1,100 × 1,100 × 1,100 voxels was selected from the central part of the soil cores, representing a volume of 1.1 × 1.1 × 1.1 cm^3^. The total porosity and pore size distribution were analysed with the “Friction Volume” and “Thickness” plugins. Pore sizes obtained from “Thickness” were expressed as the equivalent diameter. Pores were classified with a 20‐μm interval diameter from 20 to 500 μm.

### 2.7 Statistical analysis

For the comparison of pore size distribution obtained by X‐ray CT scanning, porosity was modelled using “Gaussian” regression with logarithmically (base 10) transformed soil pore size as the explanatory variate, porosity as the response variate, and treatments of distilled water and root exudates as grouping factors. The curves were fitted to data with grouped regression. We used Genstat (VSN Int. Ltd, Hemel Hempstead, UK, or Payne, 2015) to fit the curves described above to our data and for the statistical analysis.

## 3 RESULTS

### 3.1 Soil penetrometer resistance

**FIGURE 1:**
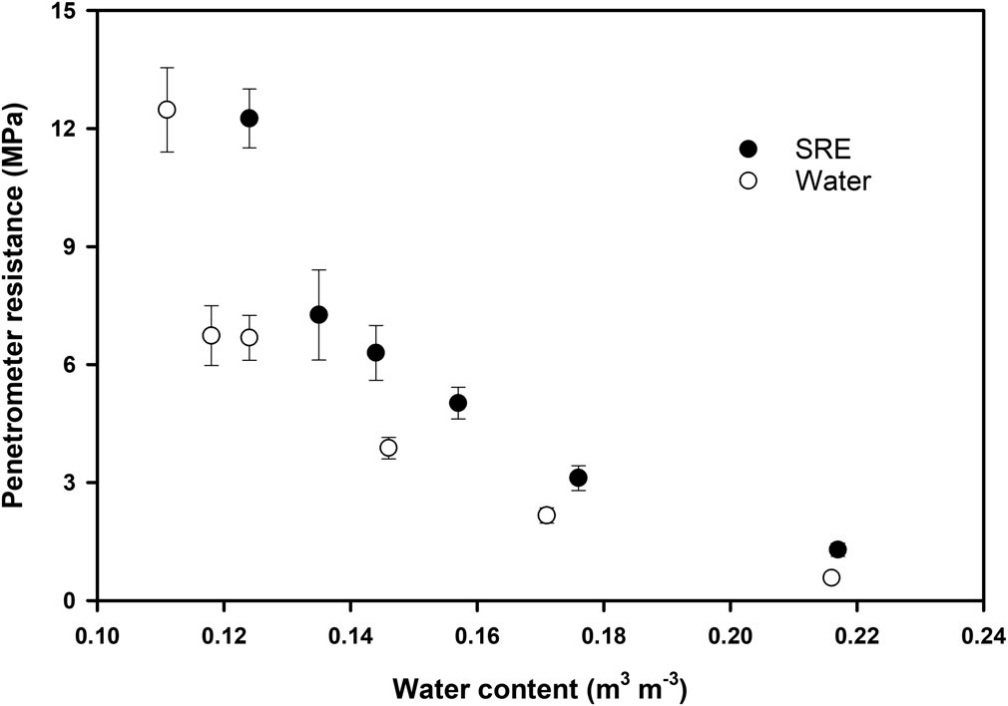
Dynamics of penetrometer resistance of synthetic root exudate (SRE)‐ and water‐treated soil samples during a drying process following incubation at 18°C

Figure 1 shows the measurements of soil PR at different water contents during the drying process. Under both SRE and water treatments, there was an exponential increase in PR with decreasing water content, and addition of root exudate increased PR at a specific water content (*p* < .05). Furthermore, the difference between SRE‐treated and water‐treated samples became larger with decreasing water content. For example, when the evaporation experiment was initiated right after incubation, both root exudate and water treatments had a water content of 0.22 m^3^ m^−3^, and the PR of SRE‐ and water‐treated samples was 1.29 MPa and 0.58 MPa, respectively; when the soil water content was reduced to about 0.12 m^3^ m^−3^ (i.e., day 5 for water treatment and day 6 for SRE treatment), the PR of SRE‐treated samples was 5.52 MPa larger than that of water‐treated samples. Thus, incubation with the SRE increased PR, and the effect was especially noticeable under dry conditions.

### 3.2 Soil thermal conductivity

**FIGURE 2:**
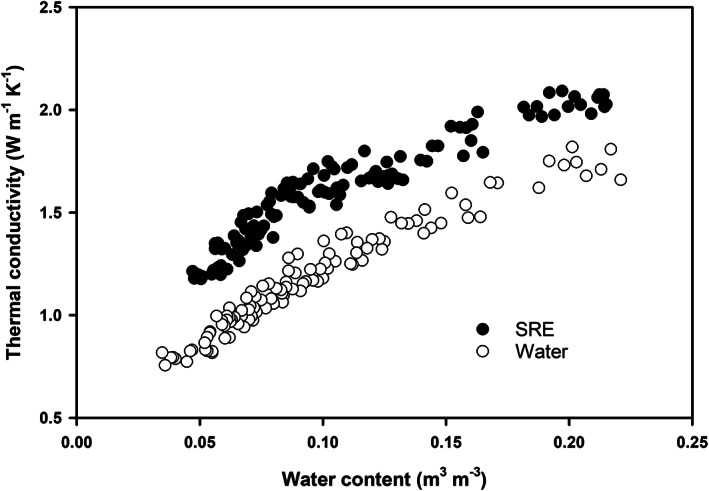
Dynamics of thermal conductivity of synthetic root exudate (SRE)‐ and water‐treated soil samples during a drying period following incubation at 18°C

Figure 2 presents the λ(θ) curves under both SRE and water treatments. The λ values on the first day were obtained right after incubation at 18°C, and the remaining values were daily measurements during the 8‐day evaporation process. For both SRE and water treatments, λ decreased nonlinearly with soil drying, and the rate of λ change showed two distinct stages: a slow λ change appeared at soil water contents greater than 0.1 m^3^ m^−3^, and a relatively rapid reduction at water contents smaller than 0.1 m^3^ m^−3^. At a specific water content, SRE‐treated samples had significantly higher λ values than those of water‐treated samples. In the 0.05–0.21 m^3^ m^−3^ water content range, for example, the SRE‐treated sample had a λ range of 1.2–2.1 W m^−1^ K^−1^ and the corresponding λ range of water‐treated samples was 0.8–1.8 W m^−1^ K^−1^. The λ difference between the two treatments was reduced slightly in the later stages of drying.

### 3.3 Soil water evaporation

**FIGURE 3:**
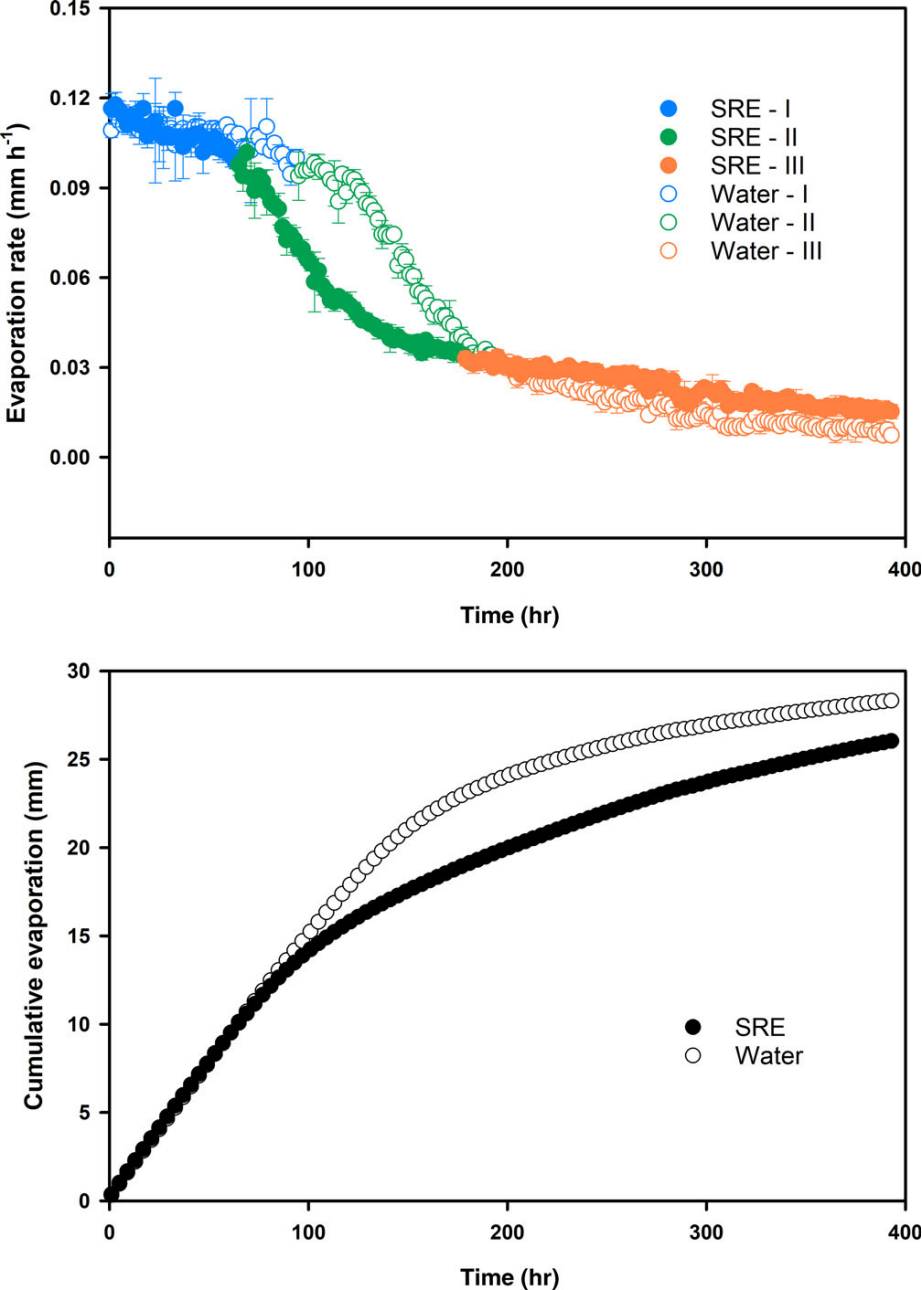
Changes of soil water evaporation rates (a) and cumulative evaporation (b) of synthetic root exudate (SRE)‐ and water‐treated samples during a drying process. The samples were incubated at 18°C for 7 days, saturated with water and then dried at room temperature

The soil drying process usually involves three stages of water evaporation: a relatively constant rate stage, a falling rate stage and a residual stage (Amano & Salvucci, 1999; Suleiman & Ritchie, 2003). Addition of the SRE changed soil water evaporation at all three stages (Figure 3a). In stage I, both SRE‐ and water‐treated samples had similar evaporation rates (about 0.10–0.12 mm h^−1^) but the duration of SRE‐treated samples was reduced to 64 h from 94 h of water‐treated ones. In stage II, evaporation rates of both SRE‐ and water‐treated samples were reduced significantly, but the samples with SRE had a lower rate of reduction and a relatively longer duration (1 hr) than the samples with water. In stage III when both treatments had low and relatively stable evaporation rates, the values (0.034–0.015 mm h^−1^) of SRE‐treated samples were slightly higher than those (0.034–0.007 mm h^−1^) of water‐treated ones.

The SRE‐ and water‐treated samples had similar values of cumulative evaporation in the 0–6‐hr period, but after 6 hr SRE‐treated samples had lower cumulative evaporation than that of water‐treated samples (Figure 3b). At the end of the evaporation study (39 hr), the cumulative evaporation values of the SRE‐ and water‐treated samples were 26.02 and 28.31 mm, respectively.

### 3.4 Soil hydraulic conductivity

**FIGURE 4:**
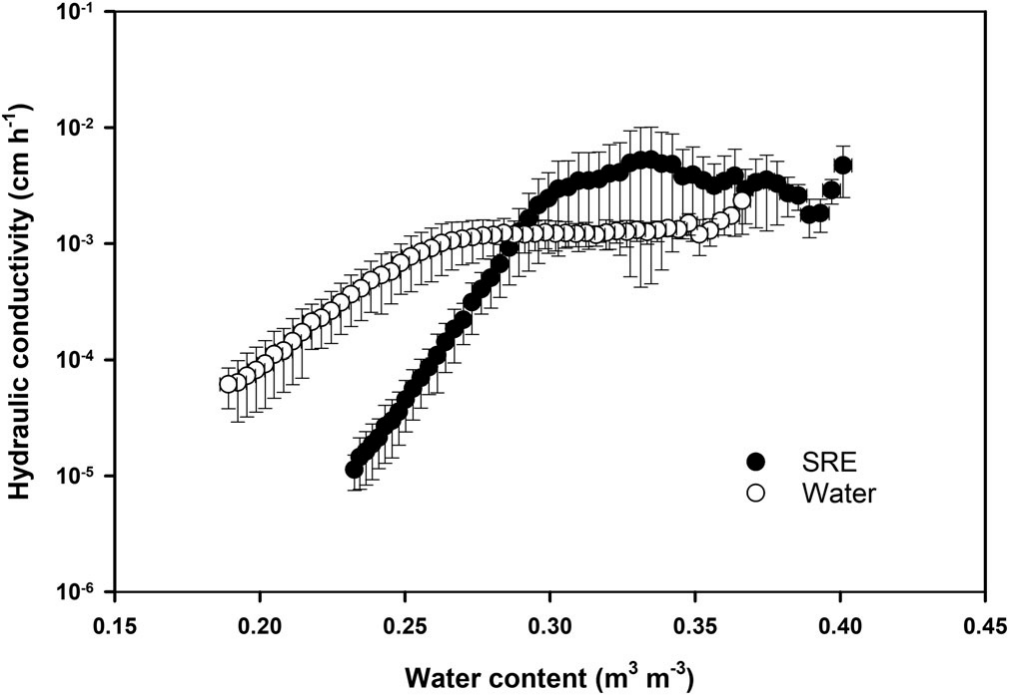
Soil hydraulic conductivity as a function of water content for synthetic root exudate (SRE)‐ and water‐treated samples obtained with the ku‐pF apparatus

Figure 4 presents the results of soil hydraulic conductivity as a function of water content (θ). For both treatments, the *k*(θ) curves could be divided into two parts: a relatively flat section with greater *k* values at higher water contents and a steep section with *k* values decreased with water content. The SRE treatment altered the shape of the *k*(θ) curve significantly. (i) The SRE treatment had a greater θ value (0.30 m^3^ m^−3^) than that of the water‐treated samples. (ii) In the flat section, SRE‐treated samples had higher hydraulic conductivities than the water‐treated samples. (iii) In the steep section, a greater falling rate of soil hydraulic conductivity was observed with the SRE‐treated samples, and its *k* values were lower than those of the water‐treated samples at θ < 0.28 m^3^ m^−3^.

### 3.5 Soil pore‐size distribution

**FIGURE 5:**
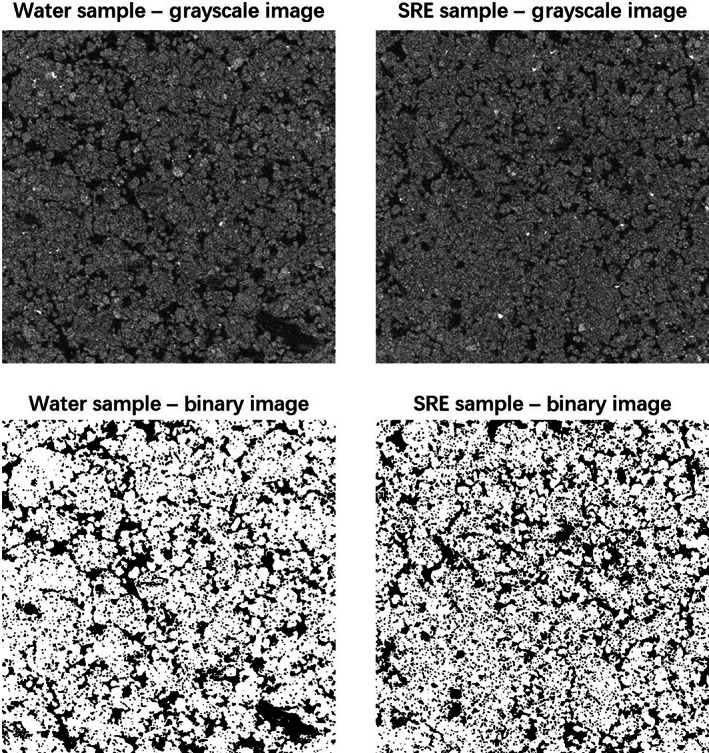
Greyscale and binary images of synthetic root exudate (SRE)‐ and water‐treated soil samples following incubation at 18°C for 7 days. The images were obtained using an X‐ray μ‐CT (computed tomography) scanner with a 10‐μm resolution. Images are displayed as 1,100 × 1,100 pixels

**FIGURE 6:**
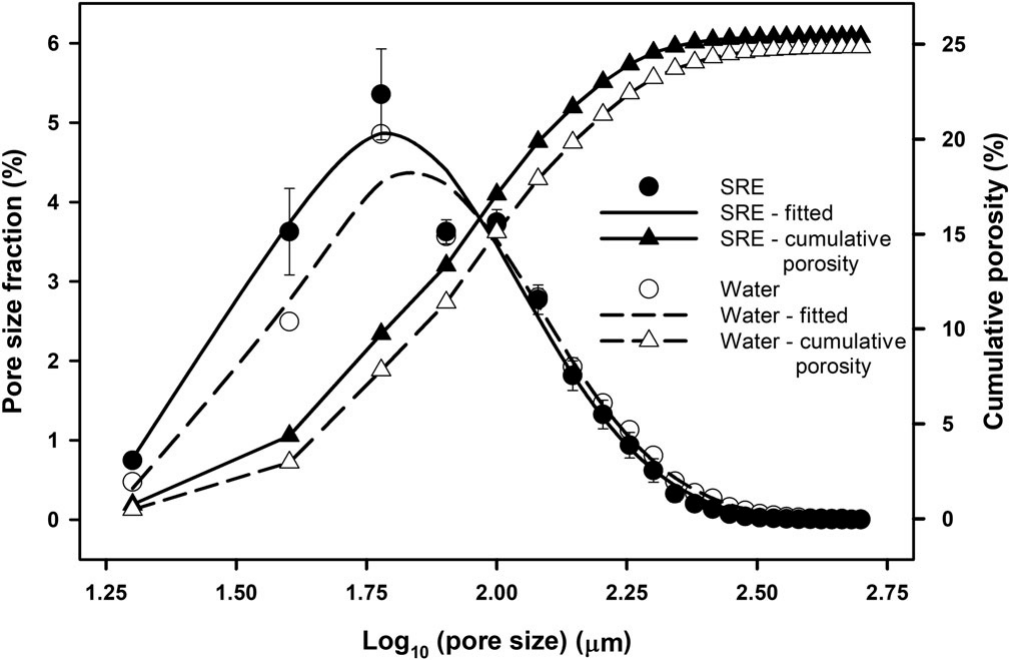
Pore size distribution of synthetic root exudate (SRE)‐ and water‐treated soil samples following incubation at 18°C for 7 days. The porosity data were estimated indirectly from X‐ray computed tomography (CT) scanned images. The values of the *x*‐axis represent logarithmic results of pore diameters

Figure 5 presents the greyscale and binary images of SRE‐ and water‐treated samples from X‐ray CT scanning. Visual observation indicated that the images of water‐treated samples had a greater number of larger pores, whereas the images of SRE‐treated samples showed a greater number of smaller pores. Pore size distribution data from X‐ray CT images are shown in Figure 6. The curves shown were fitted by using grouped regression. We used the Genstat function PRNORMAl (x, m, v), which is a probability density function for a normal distribution with mean, m, and variance, v. The fit accounted for 97.4% of the total variance in the data. Due to the limitations of image resolution, sample size and uniformity, we only calculated soil pores with diameters ranging from 20 to 500 μm. Although the two treatments had similar total porosity (25.4 vs. 24.8%, obtained from pore size distribution by the “Thickness” plugin), the SRE‐treated samples had a greater proportion of smaller pores (<60 μm) and a relatively lower portion of larger pores (>140 μm). In the pore size range of 20–60 μm, for example, the cumulative porosities were 9.73 and 7.82% for SRE‐treated and water‐treated samples, respectively. In the pore size range of 140–500 μm, the SRE‐treated samples had a cumulative porosity of 5.53%, slightly lower than that (6.94%) of the water‐treated samples. Thus, SRE incubation significantly altered soil pore size distribution.

## 4 DISCUSSION

Our results support earlier findings that, in general, addition of synthetic or natural root exudates increases soil strength (Gao et al., 2017) and reduces unsaturated soil hydraulic conductivity (Zheng et al., 2018) and soil water evaporation (Choudhury et al., 2018). These changes may relate to the properties of the SRE solution per se and SRE‐induced changes in microbial activity that have transformed the soil structure. We demonstrated that the changes of soil physical properties and processes induced by the synthetic root exudate depended on soil water status. Compared with the control samples (augmented with water), the SRE‐treated samples have greater hydraulic conductivities at higher water contents (i.e., the flat section of the *k*(θ) curve) and a relatively higher stage III evaporation rate. To the best of our knowledge, this is the first report that SRE treatment causes an increase of soil thermal conductivity.

### 4.1 Root exudates as drivers of microbial activities that promote soil structure formation

It was possible that enhanced microbial activity due to SRE incubation was responsible for the modification of soil structure and pore size distribution (i.e., a greater fraction of smaller pores and a relatively lower fraction of larger pores; Figure 5) and the subsequent changes of soil physical properties. Firstly, root exudates are sources of carbon and energy for the heterotrophic soil microflora (Morel et al., 1991). Soil microbial activity is usually stimulated by root‐released carbon, either in the form of natural root exudate (Paterson et al., 2007) or artificially prepared materials (Choudhury et al., 2018). Several studies have shown that in sandy soils, the hyphae network is mainly responsible for stabilizing soil particles into aggregates (Degens & Sparling, 1995; Degens, Sparling, & Abbott, 1996) by cross‐linkage and entanglement of particles (Degens et al., 1996; Moreno‐Espíndola, Rivera‐Becerril, de Jesús Ferrara‐Guerrero, & de León‐González, 2007; Oades, 1993; Tisdall, Nelson, Wilkinson, Smith, & McKenzie, 2012). In this study, the addition of SRE almost certainly promoted microbial activity (see Choudhury et al., 2018; Gao et al., 2017), which enhanced the soil particle‐to‐particle contacts and led to greater soil PR and λ values (Figures 1 and 2). For SRE‐treated samples, we observed that hyphae appeared on the second day of incubation (at 18°C), grew prolifically on the fourth and fifth days, and some dead or dry hyphae appeared at the end of 7 days incubation, whereas no hyphae could be seen on the water‐treated samples. By using the same SRE and similar incubation experiments, Gao et al. (2017) found increased numbers of several types of bacteria. They further showed that suppression of bacteria was more effective in increasing soil strength than suppression of fungi, suggesting the important role of fungi in shaping soil structure.

Other microorganisms might also have contributed to the structural changes of SRE‐treated samples. There are reports that bacteria and their metabolites can improve soil stability by enhancing particle‐to‐particle adhesion and affect water retention and reduce soil water diffusivity by blocking smaller pores and altering the surface tension of the water (Benard et al., 2017; Choudhury et al., 2018; Or, Phutane, & Dechesne, 2007; Papadakos et al., 2017).

### 4.2 Synthetic root exudate as a bonding agent

Root exudates usually consist of a group of organic compounds (e.g., polysaccharides, organic acids, glucose and sugars) that are generally viscoelastic and thus cause changes in soil interparticle contacts, mechanical stability (Naveed et al., 2017) and hydrological processes (Carminati, Zarebanadkouki, Kroener, Ahmed, & Holz, 2016). The anionic forms of organic acids in root exudate may disperse soil particles (Shanmuganathan & Oades, 1983), whereas the sugars offset this effect by gelling soil particles together (Oades, 1984). Polygalacturonic acid, a compound that can be used to simulate root exudates, improved the interparticle bond energy, and as a result, the fracture toughness of clay was increased exponentially with added PGA (Zhang et al., 2008). In addition, some compounds in root exudates are powerful surfactants, which decrease the surface tension at the gas–liquid surface and change soil water retention properties immediately (Raaijmakers, de Bruijn, Nybroe, & Ongena, 2010; Read et al., 2003).

It is hardly possible to differentiate the bonding effects of the SRE from microbial‐induced soil structural changes. For this purpose, we compared the PR and λ values in this study against measurements from another experiment conducted at 4°C, in which microbial activity was inhibited and the changes of soil physical properties were supposed to be caused merely by the SRE as a bonding agent. Paired (SRE‐treated and water‐treated) samples were prepared with the same procedure, except that the samples were incubated at 4°C for 7 days. At the end of incubation, the SRE‐treated samples had a higher PR (974 kPa) than that of the water‐treated samples (634 kPa, Figure S1). The difference, however, was much smaller compared with the results from 18°C incubation (1,331 kPa vs. 597 kPa). Additionally, the λ values of SRE‐treated samples (2.06 W m^−1^ K^−1^) showed no significant difference from those of water‐treated samples incubated either at 4°C (1.94 W m^−1^ K^−1^) or at 18°C (2.08 W m^−1^ K^−1^, Figure S1). Thus, the SRE used in this study could have promoted bonding at low temperatures when the role of microorganisms was minimized. At normal temperatures (e.g., 18°C), the direct “sticking effect” of the SRE is minor compared to its indirect function of simulating microbial activity.

### 4.3 Effects of synthetic root exudate on soil evaporation and hydraulic conductivity

The influence of root exudates and similar materials (e.g., EPS produced by microbes) on soil hydraulic conductivity, water retention and evaporation has been studied extensively. In general, the addition of root exudates and EPS leads to a reduction of hydraulic conductivity (Or et al., 2007; Vandevivere & Baveye, 1992), increase of water retention and a decline of evaporative water loss (Chenu & Roberson, 1996; Choudhury et al., 2018). These phenomena are explained by the experimental evidence that these materials have a larger water holding capacity, alter the soil matrix structure and connectivity of pore space, and modify the surface tension and viscosity of soil water (Naveed et al., 2018; Zheng et al., 2018). Our results support the previous findings, in that the SRE‐treated samples had lower average evaporation rates and reduced cumulative evaporative water losses than the water‐treated samples (Figure 3). However, the two treatments differed considerably in terms of duration and rate of evaporation at the three stages. During stage I evaporation, both treatments had a relatively high and constant evaporation rate controlled by atmospheric conditions. Yet the SRE‐treated samples had a shorter stage I period, because the soils were not able to sustain liquid water flow to meet the evaporative demand due to the sudden reduction of hydraulic conductivity at the inflection point (Figure 4). Additionally, because they had a greater water holding capacity and lower hydraulic conductivity than the water‐treated samples, the SRE‐treated samples maintained a longer but slower water loss rate in stage II evaporation. It was surprising that the SRE‐treated samples had a relatively higher rate of stage III evaporation than the water‐treated ones, which has not been reported previously. This is likely to be because the SRE‐treated samples maintained higher water contents (mostly absorbed water) at the end of stage II evaporation.

Interestingly, our data showed that addition of the SRE did not always reduce soil hydraulic conductivity. Compared to the water‐treated samples, elevated hydraulic conductivities were obtained in the SRE‐treated samples in the flat section of the *k*(θ) curve (Figure 4). We are not clear about the explanation for this phenomena because both treatments had similar total porosities and the SRE treatment possessed a greater proportion of smaller pores (Figures 5 and 6). A potential explanation is that some preferential flow paths had been created due to SRE‐induced microbial activities.

## 5 CONCLUSIONS

In this study, we investigated the mechanical strength (penetrometer resistance), thermal conductivity, hydraulic conductivity and evaporation process of a sandy soil treated with a synthetic root exudate. After incubation at 18°C, both penetrometer resistance and thermal conductivity were increased, soil hydraulic conductivity was increased in the wet region but decreased in the dry region, and overall evaporation rate and cumulative water loss were reduced significantly, which was attributed to reinforced particle‐to‐particle contacts and changes of soil pore size distribution due mainly to exudate‐stimulated microbial activity. X‐ray CT scanning images provided direct evidence that root exudate treatment had little effect on total porosity but increased the number of smaller pores. Further studies are required to examine if these conclusions also apply to field soils with natural root exudates.

## AUTHOR CONTRIBUTIONS

WZ conducted the experiment, collected the data and drafted the article. WG, WRW and TR participated in the design of the work, helped with data analysis and interpretation and revised the manuscript. All authors approved the final version to be published.

## CONFLICT OF INTERESTS

The authors declare that there is no conflict of interests regarding the publication of this article.

## Supporting information


**Appendix S1:** Supplementary materials

**Figure S1** Comparison of penetrometer resistance (a) and thermal conductivity (b) of synthetic root exudate‐treated (SRE) samples and distilled water‐treated (DW) samples of a sandy soil incubated at 4 and 18°C, respectively.

## Data Availability

The data that support the findings of this study are available from the corresponding author upon reasonable request.
