# Supplementary material for: Physical properties of a sandy soil as affected by incubation with a synthetic root exudate: Strength, thermal and hydraulic conductivity, and evaporation
Source: Eur J Soil Sci. 2020 Jun 28;72(2):782–92. doi: 10.1111/ejss.13007 (PMC7984329; doi:10.1111/ejss.13007)
Supplement: Supplementary file 1 — Appendix S1: Supplementary materials [file EJSS-72-782-s001.docx]

**Supplementary materials**

1. **Temperature dependence of the performance of synthetic root exudate**

A set of synthetic root exudate (SRE)-treated and water-treated soil columns (50-mm high and 50-mm I.D.) were prepared following the same procedure but were incubated at 4^o^C for 7 days. The assumption is that at a temperature of 4^o^C, soil microbial activity is inhibited, and the changes of soil physical properties are caused merely by SRE as a bonding agent. At the end of incubation, the SRE-treated sample had a penetrometer resistance (PR) of 974 kPa, higher than that of the water-treated samples (634 kPa). The thermal conductivity (λ) value of the SRE-treated sample (2.06 W m^-1^ K^-1^), on the other hand, did not diff significantly from that of the water-treated sample (1.94 W m^-1^ K^-1^). Additionally, both λ values obtained at 18^o^C did not differ significantly from that of water-treated samples incubated at 18^o^C (2.08 W m^-1^ K^-1^).


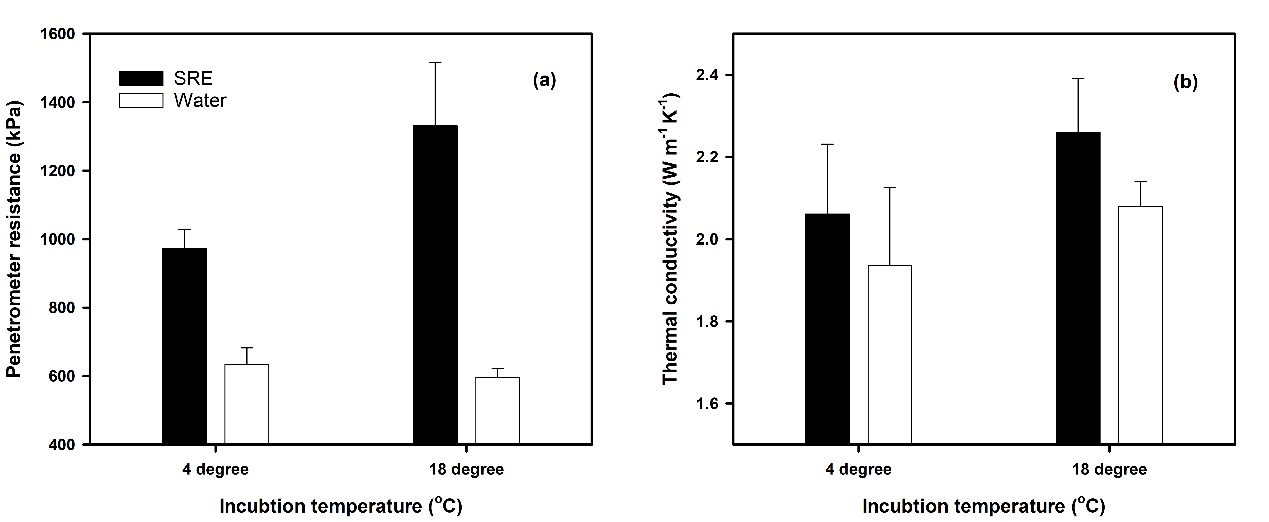


**Figure S1** Comparison of penetrometer resistance (a) and thermal conductivity (b) of synthetic root exudate-treated (SRE) samples and distilled water-treated (DW) samples of a sandy soil incubated at 4 and 18^o^C, respectively.
