# Supplementary figures and images for: Physical properties of a sandy soil as affected by incubation with a synthetic root exudate: Strength, thermal and hydraulic conductivity, and evaporation
Source: Eur J Soil Sci. 2020 Jun 28;72(2):782–92. doi: 10.1111/ejss.13007 (PMC7984329; doi:10.1111/ejss.13007)

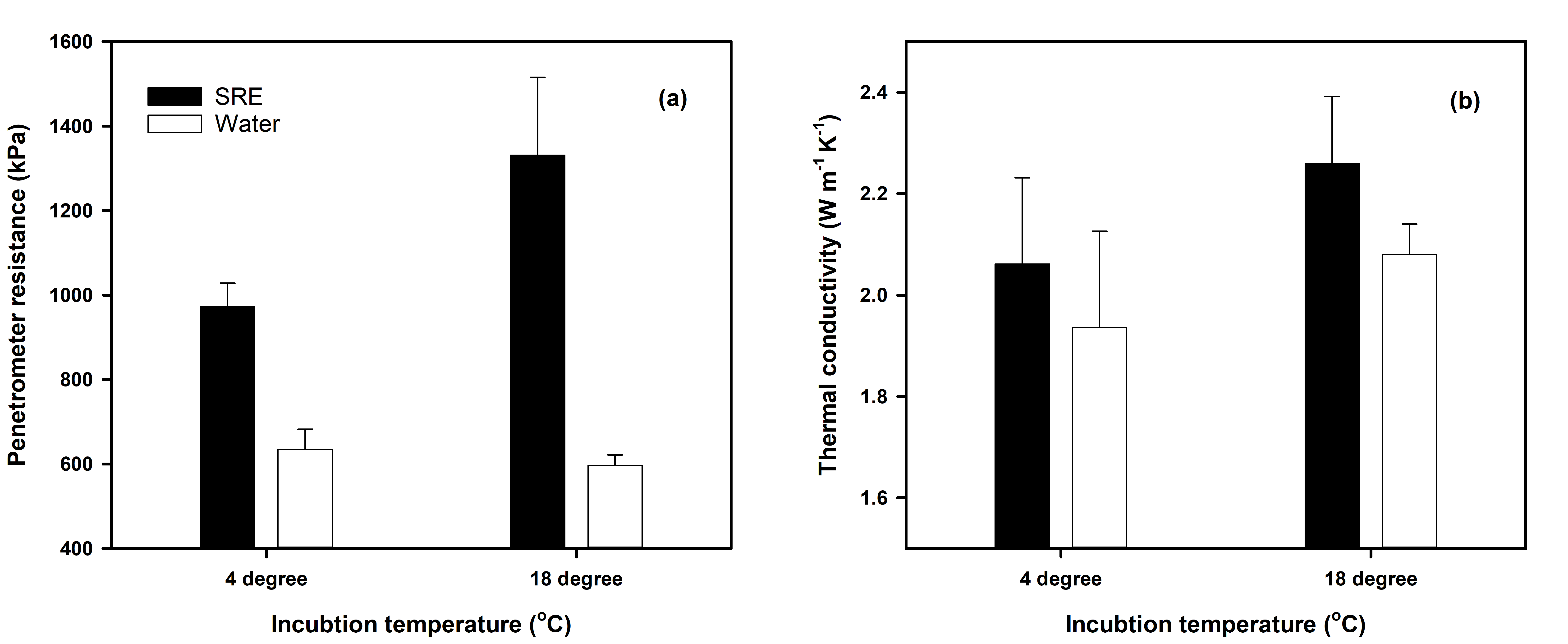

Supplement: Supplementary file 2 — Figure S1 Comparison of penetrometer resistance (a) and thermal conductivity (b) of synthetic root exudate‐treated (SRE) samples and distilled water‐treated (DW) samples of a sandy soil incubated at 4 and 18°C, respectively. [file EJSS-72-782-s002.TIF]
